# Mucosal Prevalence and Interactions with the Epithelium Indicate Commensalism of *Sutterella* spp.

**DOI:** 10.3389/fmicb.2016.01706

**Published:** 2016-10-26

**Authors:** Kaisa Hiippala, Veera Kainulainen, Marko Kalliomäki, Perttu Arkkila, Reetta Satokari

**Affiliations:** ^1^Immunobiology Research Program, Faculty of Medicine, University of HelsinkiHelsinki, Finland; ^2^Pharmacology, Faculty of Medicine, University of HelsinkiHelsinki, Finland; ^3^Department of Pediatrics, Turku University Central Hospital and Functional Foods Forum, University of TurkuTurku, Finland; ^4^Department of Gastroenterology, Helsinki University Central HospitalHelsinki, Finland

**Keywords:** *Sutterella*, host–microbe interaction, adherence, gut inflammation, commensal, Proteobacteria

## Abstract

*Sutterella* species have been frequently associated with human diseases, such as autism, Down syndrome, and inflammatory bowel disease (IBD), but the impact of these bacteria on health still remains unclear. Especially the interactions of *Sutterella* spp. with the host are largely unknown, despite of the species being highly prevalent. In this study, we addressed the interaction of three known species of *Sutterella* with the intestinal epithelium and examined their adhesion properties, the effect on intestinal barrier function and the pro-inflammatory capacity *in vitro*. We also studied the relative abundance and prevalence of the genus *Sutterella* and *Sutterella wadsworthensis* in intestinal biopsies of healthy individuals and patients with celiac disease (CeD) or IBD. Our results show that *Sutterella* spp. are abundant in the duodenum of healthy adults with a decreasing gradient toward the colon. No difference was detected in the prevalence of *Sutterella* between the pediatric IBD or CeD patients and the healthy controls. *Sutterella parvirubra* adhered better than the two other *Sutterella* spp. to differentiated Caco-2 cells and was capable of decreasing the adherence of *S. wadsworthensis*, which preferably bound to mucus and human extracellular matrix proteins. Furthermore, only *S. wadsworthensis* induced an interleukin-8 production in enterocytes, which could be due to different lipopolysaccharide structures between the species. However, its pro-inflammatory activity was modest as compared to non-pathogenic *Escherichia coli*. *Sutterella* spp. had no effect on the enterocyte monolayer integrity *in vitro*. Our findings indicate that the members of genus *Sutterella* are widely prevalent commensals with mild pro-inflammatory capacity in the human gastrointestinal tract and do not contribute significantly to the disrupted epithelial homeostasis associated with microbiota dysbiosis and increase of Proteobacteria. The ability of *Sutterella* spp. to adhere to intestinal epithelial cells indicate that they may have an immunomodulatory role.

## Introduction

The three known species of the genus *Sutterella*, consisting of *Sutterella wadsworthensis*, *Sutterella parvirubra*, and *Sutterella stercoricanis*, have been isolated from the human gastrointestinal (GI) tract and canine feces (Wexler et al., 1996; Greetham et al., 2004; Sakon et al., 2008). *Sutterella* spp., belonging to Betaproteobacteria, are Gram-negative, non-spore-forming rods that grow in a microaerophilic atmosphere or under anaerobic conditions (Wexler, 2005). They are notably resistant to human bile acids, which may account for their survival in the biliary tract and bowel. The role of *Sutterella* spp. as part of the human microbiota is largely unexplored with published reports being partly controversial. The lack of knowledge is rather surprising since *Sutterella* spp. have been reported to be highly prevalent and abundant in the intestinal mucosa of humans (Mukhopadhya et al., 2011; Cheng et al., 2013; Hansen et al., 2013). Firstly, *S. wadsworthensis* has been detected in 86 and 71% of adults and children, respectively (Mukhopadhya et al., 2011; Hansen et al., 2013). Secondly, the average abundance of *Sutterella* spp. has been found to be as high as 19% of the total microbiota in the duodenal mucosa of both celiac disease (CeD) and healthy children (Cheng et al., 2013).

Although *Sutterella* spp. have been suspected to play a part in the pathogenesis of inflammatory bowel disease (IBD; Mangin et al., 2004; Gophna et al., 2006; Lavelle et al., 2015), in other studies no difference in the prevalence of *Sutterella* spp. has been found between the IBD patients and the healthy subjects (Mukhopadhya et al., 2011; Hansen et al., 2013). Furthermore, *Sutterella* spp. have been associated with autism, Down syndrome, and metabolic syndrome (Williams et al., 2012; Wang et al., 2013; Biagi et al., 2014; Lim et al., 2016). Half of the children with autism and GI dysfunction were *Sutterella*-positive with a predominance of either *S. wadsworthensis* or *S. stercoricanis*, whereas these species were not detected in the control group with only GI dysfunction (Williams et al., 2012). The abundance varied between 2 and 7% of total bacteria in the ileum and cecum. However, in another study no significant difference in the microbial composition, including the abundance of *Sutterella*, was observed between the autistic pediatric subjects with or without functional GI disorders compared to their siblings (Son et al., 2015). Thus, the results concerning the association of *Sutterella* spp. with human diseases are controversary and their possible role in the pathology and interaction with the host are still unknown.

Commensals form a diverse and balanced bacterial community, which colonizes the mammalian mucosal surfaces without causing harm to the host. As part of the host–microbiota mutualism, commensal bacteria shape the development of both innate and adaptive immune systems and promote immunological tolerance in the host (Clemente et al., 2012; Nicholson et al., 2012). Various pattern recognition receptors on intestinal epithelial cells (IECs), such as Toll-like receptors (TLRs), recognize microorganism-associated molecular patterns (MAMPs) expressed by bacteria (Round et al., 2011; Bron et al., 2012). A major MAMP produced by Gram-negative bacteria on their cell surface is lipopolysaccharide (LPS), which activates TLR4 signaling, thus triggering inflammatory cascades (Miller et al., 2005). IECs can sense MAMPs and consequently act as transmitters of immunomodulatory signals from the microbiota to immune cells in lamina propria (LP), where most of the immune cells do not have direct contact with bacteria (de Kivit et al., 2011; Ivanov and Honda, 2012). Specifically, gut commensals are needed to induce the differentiation of Th17 cells in LP (Gaboriau-Routhiau et al., 2009; Ivanov et al., 2009; Atarashi et al., 2015) and importantly, this stimulation is dependent on the adhesion of bacteria to IECs (Atarashi et al., 2015).

The interaction between a balanced, stable microbiota and the mucosal immune system maintains intestinal homeostasis, which, however, can be easily disturbed by alterations in the microbial community leading to pro-inflammatory immune responses. This imbalance in the composition of gut microbiota (dysbiosis) is generally characterized by a decrease in the abundance of Firmicutes and an increase of Proteobacteria, the phylum that also contains *Sutterella* spp. (Mukhopadhya et al., 2012; Shin et al., 2015). In a healthy GI tract the relative abundance of Proteobacteria varies between 2 and 5%, whereas in metabolic disorders and intestinal inflammation the proportion can be up to 15% (Fei and Zhao, 2013; Gevers et al., 2014; Walujkar et al., 2014; Lavelle et al., 2015; Shin et al., 2015). Increase in the amount of Proteobacteria may contribute to non-specific mucosal inflammation due to LPS as a potent stimulator and possibly predispose the host to a chronic inflammatory disease (Round and Mazmanian, 2009). Therefore, it is important to determine the interactions and pro-inflammatory capacity of different representative taxa of Proteobacteria and establish in what scale they play a part in the microbiota dysbiosis and deleterious effects associated with it.

The epithelial interactions and the role of *Sutterella* spp. in the human GI tract still remain unclear. In this study, we addressed the adhesive and pro-inflammatory properties of three known *Sutterella* spp. using various *in vitro* models, including the adhesion to intestinal epithelial cell lines, mucus and extracellular matrix (ECM) proteins, as well as their effect on the epithelial barrier function and interleukin-8 (IL-8) release in enterocytes. In addition, we investigated the relative abundance of these species in different parts of the human intestine and the prevalence in pediatric patients with CeD or IBD.

## Materials and Methods

### Bacterial Strains and Growth Conditions

*Sutterella wadsworthensis* (DSM 14016 = ATCC 51579, type strain), *S. parvirubra* (DSM 19354 = YIT 11816^T^, type strain) and *S. stercoricanis* (DSM 17807 = CCUG 47620^T^, type strain) were obtained from German Collection of Microorganisms and Cell Cultures (DSMZ) and grown on Gifu anaerobic medium (GAM; Nissui Pharmaceutical Co., Ltd., Japan) agar for 2 days at 37°C in an anaerobic incubator (Concept Plus anaerobic workstation, Ruskinn Technology Ltd., UK) containing 85% N_2_, 10% CO_2_, and 5% H_2_. *Bacteroides fragilis* E-022248^T^ received from the VTT Culture Collection (VTT Technical Research Center of Finland) was grown under anaerobic conditions for 2 days at 37°C on Brucella agar with hemin and vitamin K (Fluka, USA) along with 5% defibrinated sheep blood (Bio Karjalohja Oy, Finland). *Escherichia coli* K12-derived TOP10 cells (Invitrogen, USA) were aerobically cultivated in Luria–Bertani broth (Becton Dickinson, USA) overnight at 37°C with agitation (220 rpm). For adhesion experiments, *Sutterella* spp. were grown on GAM agar supplemented with 10 μl ml^-1^ of [6′-^3^H]thymidine (17,6 Ci mmol^-1^, Perkin Elmer, USA) to metabolically radiolabel the cells.

### Epithelial Cell Lines

The human colonic epithelial cell lines Caco-2 (ACC 169) and HT-29 (ACC 299) were obtained from DSMZ and grown at 37°C in an incubator under oxic atmosphere with 5% CO_2_. Cells were passaged after reaching 80% confluence using HyClone HyQTase (GE Healthcare Life Sciences, USA) to detach the cells. Passage numbers 5–28 were used in the experiments. Caco-2 cells were grown in RPMI 1640 medium (Sigma-Aldrich, USA) supplemented with heat-inactivated (30 min at 56°C) fetal bovine serum (FBS, 20%; Integro B.V., Netherlands), non-essential amino acids (1%; Lonza, Belgium), 15 mM HEPES (Lonza, Belgium), 100 U ml^-1^ penicillin and streptomycin (PEST; Lonza, Belgium), and 2 mM L-glutamine (Lonza, Belgium). HT-29 cells were cultivated in McCoy 5A (Lonza, Belgium) medium containing 10% FBS and 100 U ml^-1^ PEST. For adhesion and ELISA assays, 10,000 Caco-2 or HT-29 cells per well were seeded onto 96-well microplates.

### Isolation of Human Intestinal Mucus

Human colonic mucus was isolated from a healthy piece of tissue from a colorectal cancer patient as previously described (Vesterlund et al., 2005). The use of human intestinal mucus for the adhesion assays was approved by the ethical committee of the Hospital District of Southwest Finland. A written informed consent was obtained from all patients who donated tissue.

### Bacterial DNA Extraction

*Sutterella* spp. and *B. fragilis* bacterial masses were collected from agar plates with an inoculation loop and suspended to lysis buffer. *E. coli* broth culture was centrifuged (5,000 × *g*, 5 min) to pellet the cells and DNA was extracted with QIAamp DNA mini kit (Qiagen, UK) according to the manufacturer’s instructions. DNA concentrations were measured using NanoDrop ND-1000 spectrophotometer (NanoDrop Technologies, USA).

### Study Subjects and Clinical Samples

Intestinal mucosal biopsies were obtained from adult volunteers recruited at the Department of Gastroenterology at Helsinki University Central Hospital (5 males and 17 females, mean age 65 years), who underwent diagnostic nasogastroduodenoscopy or/and colonoscopy and were found to have a healthy intestine (Supplementary Table S1). Rectal biopsies were also obtained from adult patients (four males and seven females, mean age 57 years) who had been successfully treated with a fecal microbiota transplantation (FMT) for recurrent *Clostridium difficile* infection (rCDI; Supplementary Table S1; Satokari et al., 2015). Biopsies were collected in sterile tubes and stored at -80°C until the DNA extraction. DNA from the biopsy samples was extracted as described previously (Kalliomaki et al., 2012), involving a mechanical lysis step for the efficient recovery of bacterial DNA. The study was approved by the Ethics Committee of Hospital District of Helsinki and Uusimaa (HUS), Finland. All healthy adult volunteers who donated biopsies signed an informed consent.

Intestinal mucosal biopsies from pediatric patients were obtained from our previous studies (Kalliomaki et al., 2012, 2014) or newly collected at the Department of Pediatrics in Turku University Central Hospital. DNA was isolated as mentioned above. The study subjects included healthy controls (*n* = 33) and patients with CeD (*n* = 9), Crohn’s disease (CD, *n* = 14), and ulcerative colitis (UC, *n* = 25). The age and gender of the subjects, the location of biopsies, and disease activity are compiled in Supplementary Table S2. The study was approved by the Ethics Committee of the Hospital District of Southwest Finland. A written informed consent was obtained from all of the study patients and/or their parents.

### Quantitative Real-Time PCR Assays

Quantification of *Sutterella* spp. and *S. wadsworthensis* from the intestinal biopsy samples was carried out using previously described quantitative real-time PCR (qPCR) assays (Suzuki and Giovannoni, 1996; Paliy et al., 2009; Mukhopadhya et al., 2011; Williams et al., 2012). The standard DNA for *Sutterella* genus assay was prepared from amplified 260-bp region between V6 and V8 of the 16S rRNA gene from *S. wadsworthensis* (ATCC 51579). Primers were SuttFor (5′-CGC GAA AAA CCT TAC CTA GCC-3′) and SuttRev (5′-GAC GTG TGA GGC CCT AGC C-3′) (Williams et al., 2012). For *S. wadsworthensis* specific assay standard, a 555-bp long PCR-fragment of the 16S rRNA gene from *S. wadsworthensis* was amplified using forward primer SWF (5′-GAC GAA AAG GGA TGC GAT AA-3′) and reverse primer SWR (5′-CTG GCA TGT CAA GGC TAG GT-3′) (Mukhopadhya et al., 2011). The standard DNA for universal assay was produced by amplifying a 310-bp long fragment of the 16S rRNA gene from *Prevotella melaninogenica* using universal bacterial primers 27F-DegL (5′-AGR GTT YGA TYM TGG CTC AG-3′) (Paliy et al., 2009) and 338R (5′-TGC TGC CTC CCG TAG GAG T-3′) (Suzuki and Giovannoni, 1996). All the PCR-fragments were purified using QIAquick PCR purification kit (Qiagen, Germany) and DNA concentrations were measured with NanoDrop ND-1000 spectrophotometer (NanoDrop Technologies, USA). Based on the calculated molecular mass of the amplicon, the number of copies per microliter was calculated for each PCR-fragment to create six-point standard curves using 10-fold serial dilutions (10^6^–10^1^ copies). Samples were analyzed in triplicate using a 96-well plate format. Amplification and detection of DNA by qPCR were performed with Mx3005P Real-time PCR System (Stratagene, USA) and the results were analyzed with MxPro Real-time PCR Software version 4.1 (Stratagene, USA). AmpliTaq Gold DNA Polymerase kit (Applied Biosystems, Life Technologies, USA) and SYBR Green chemistry were used. Each 25 μl reaction contained 25 ng of biopsy DNA, 0.5 μM of each primers, 0.1 mM deoxynucleoside triphosphate (dNTP) mix (Thermo Scientific, USA), 10 mM Tris–HCl (pH 8.3), 50 mM KCl, 3 mM MgCl_2_, 1:75,000 dilution of SYBR Green I (Lonza Biosciences, Switzerland), and 0.025 U μl^-1^ AmpliTaq Gold DNA Polymerase. The thermocycling parameters consisted of an initial denaturation step at 95°C for 10 min, followed by 45 cycles of 95°C (30 s), 58°C (1 min), and 72°C (1 min).

### Adhesion Assays to Cell Lines, Mucus, and ECM Proteins

The adherence of *Sutterella* spp. to Caco-2 and HT-29 cell lines (3, 8, and 21 days post-plating), mucus, and ECM proteins was studied as described previously (Kainulainen et al., 2013; Reunanen et al., 2015). Briefly, the Caco-2 and HT-29 cell lines were seeded on a 96-well tissue culture plate with 10,000 cells well^-1^. Human mucus preparations collected from several patients were pooled evenly and diluted in phosphate-buffered saline (PBS). Porcine mucus (Sigma-Aldrich) and human ECM proteins, including collagens I (Sigma-Aldrich, USA) and IV (Sigma-Aldrich), laminin (Sigma-Aldrich), and fibronectin (Calbiochem, USA), were diluted in PBS. Bovine serum albumin (BSA; Sigma-Aldrich) and fetuin (Sigma-Aldrich) were used as controls for non-specific binding. Human and porcine mucus (50 μg well^-1^ in PBS) and ECM proteins (2.5 pmol well^-1^ in PBS) were incubated on Maxisorp microtiter plates overnight at 4°C and blocked with 0.5% (w/v) BSA in PBS for 1 h prior to the adhesion assay. Four technical replicates (parallel wells) were used in each experiment. [3^H^]Thymidine-labeled *S. wadsworthensis*, *S. parvirubra*, and *S. stercoricanis* cells were collected from agar plates to PBS, washed with an appropriate medium (McCoy 5A for HT-29 cells and RPMI 1640 for Caco-2) without supplements or PBS and adjusted to OD_600__nm_ 0.25, which corresponded to approximately 10^8^ cells ml^-1^. The bacterial suspensions were incubated for 1 h on mucus and ECM proteins at 37°C or on the cell lines at 37°C in a CO_2_ incubator, followed by washing three times to remove the non-adherent cells. Bound bacteria were lysed with 1% SDS–0.1 M NaOH and radioactivity was measured with a liquid scintillator (Wallac Winspectral 1414, Perkin-Elmer, USA). The percentage of adhered bacteria was calculated relative to the radioactivity of the bacterial suspension initially added to the wells. The adhesion to ECM proteins was also calculated as percentages and compared to the level of background binding (BSA).

### Displacement, Competition, and Exclusion Assays

The displacement, exclusion, and competition assays with *S. parvirubra* and radiolabeled *S. wadsworthensis* were performed as previously described with minor modifications (Vesterlund et al., 2006). The bacteria were adjusted to OD_600__nm_ 0.25. Four technical replicates (parallel wells) were used in each experiment. Eight days post-plating Caco-2 and HT-29 cells (10,000 cells well^-1^) were fixed with 4% paraformaldehyde (Sigma-Aldrich, USA) for 2 h at room temperature before the assay. The cell monolayers were fixed due to multiple, repeated washing steps, which otherwise cause detaching of the non-fixed cells. In the competition assays, both species were incubated with the enterocytes together, whereas in the exclusion assays *S. parvirubra* was incubated first, then washed away and followed by *S. wadsworthensis*. In the displacement assays *S. wadsworthensis* adhered first followed by *S. parvirubra*. The percentage of adhered bacteria was calculated relative to the radioactivity of the bacterial suspension initially added to the wells and the non-competitive situation with similar washing steps is shown as the control.

### TER Assay

The impact of *Sutterella* spp. on transepithelial electrical resistance (TER) of Caco-2 monolayer was determined as previously described (Kainulainen et al., 2015; Reunanen et al., 2015). The Caco-2 cell line is a suitable model for TER assays, because Caco-2 cells undergo enterocytic differentiation as well as express intercellular junctional complexes and, therefore, changes in TER can be measured as a response to external stimuli (Hidalgo et al., 1989; Klingberg et al., 2005; Hsieh et al., 2015). *E. coli* TOP10 was included in the experiment as a negative control, since the bacterium has been shown to adversely affect the monolayer integrity (Reunanen et al., 2015). *B. fragilis*, on the other hand, increases TER of epithelial cells and was used as a positive control (Reunanen et al., 2015). The blank resistance (measurement at time point 0) was subtracted from the measurements made after 24 and 48 h of incubation, and the unit area resistance (Ωcm^2^) was calculated by multiplying the tissue resistance values by surface area of the filter membrane.

### IL-8 Production in HT-29 Cells

IL-8 assays were only performed using the HT-29 cell line, because Caco-2 cells are known to be unresponsive to LPS stimulation possibly due to defects in TLR4 signaling (Funda et al., 2001; Van De Walle et al., 2010; Hsu et al., 2011). The measurement of IL-8 response in 8-day-old HT-29 cells by *Sutterella* spp., *E. coli* and *B. fragilis* was carried out as previously described (Kainulainen et al., 2013). In brief, the bacterial suspensions were washed with McCoy 5A medium supplemented with 10% FBS and adjusted to OD_600__nm_ 0.25. Bacteria were diluted to 1:10, 1:100, and 1:1,000 and incubated on the HT-29 cells for 3 h at 37°C in a CO_2_ incubator. Three technical replicates (parallel wells) were used in each experiment. OptEIA Human IL-8 ELISA kit (BD Biosciences, USA) was used according to the manufacturer’s instructions to measure the concentration of the chemokine in the culture media. To prepare the LPS preparations, bacteria were adjusted to OD_600__nm_ 0.25 and 500 μl of the suspension was centrifuged (9,000 × *g*, 10 min.) to pellet the cells. The pellets were suspended to 20 μl of lysis buffer (2% SDS, 50 mM Tris–HCL) and heated to 100°C for 10 min. After cooling, proteinase K (Thermo Scientific, USA) was added to the cell lysates with a final concentration of 0.5 mg ml^-1^ and incubated at 60°C for 2 h. The lysates were once again heated to 100°C to inactive proteinase K, then 450 μl of McCoy 5A medium supplemented with 10% heat-inactivated FBS was added and the lysates were diluted to 1:10–1:10,000.

### Silver Staining of *Sutterella* LPS and Western Blotting

The LPS structures of *Sutterella* spp. and *E. coli* TOP10 were visualized with silver staining as previously described (Tsai and Frasch, 1982). Prior to SDS-PAGE the bacterial pellets were suspended to loading buffer and heated to 100°C for 5 min. The whole-cell lysates were treated with proteinase K (final concentration 0.5 mg ml^-1^) at 60°C for 2 h, followed by heat-inactivation of the enzyme. The samples were run in SDS-PAGE under denaturing conditions on a gradient gel (4–15%, Mini-Protean TGX, Bio-Rad, USA) and silver stained. Proteinase K treatment reduces bacterial proteins to small peptides but leaves the carbohydrate LPS unaffected, which then can be detected due to periodate-sensitive *cis*-glycols in the core and the side-chains of LPS (Kropinski et al., 1986). The LPS chains that have the same number of sugar repeats co-migrate and form a band. The whole-cell lysates mentioned above were also analyzed with Western blotting using a rabbit polyclonal antiserum against *E. coli* LPS (1:10,000 dilution; Bioss Inc., USA). Briefly, the bacterial pellets were run in SDS-PAGE as explained above without the proteinase K treatment and blotted onto polyvinylidene difluoride Immobilon P membranes (Millipore, USA). After the *E. coli* LPS antiserum, the membrane was incubated with 1:100,000 diluted HRP-conjugated goat anti-rabbit IgG secondary antibody (Bio-Rad, USA). Amersham ECL Advance Western blotting detection kit (GE Healthcare Bio-Sciences, UK) was used according to the manufacturer’s instructions.

### Statistical Analysis

All the experiments were done using three to four technical replicates depending on the assay and repeated two to three times (biological replicates) to confirm the results. Different cultures of bacteria and different passages of cell lines were used in the separate experiments. The following tests were used to compare samples within an experiment. A two-sample *t*-test was used to determine significant differences as compared to the control. Homoscedasticity testing was performed with Levene’s test to identify equal or unequal variances. A paired-sample *t*-test was used for pre- and post-FMT samples. Statistical differences between the samples were tested with one-way analysis of variance using a Bonferroni *post hoc*. All statistical analyses were carried out with IBM SPSS Statistics program version 21.0 (IBM Corporation, USA). A *p*-value of <0.05 was considered statistically significant.

## Results

### A Decreasing Gradient of *Sutterella* spp. Abundance along the GI Tract

**FIGURE 1:**
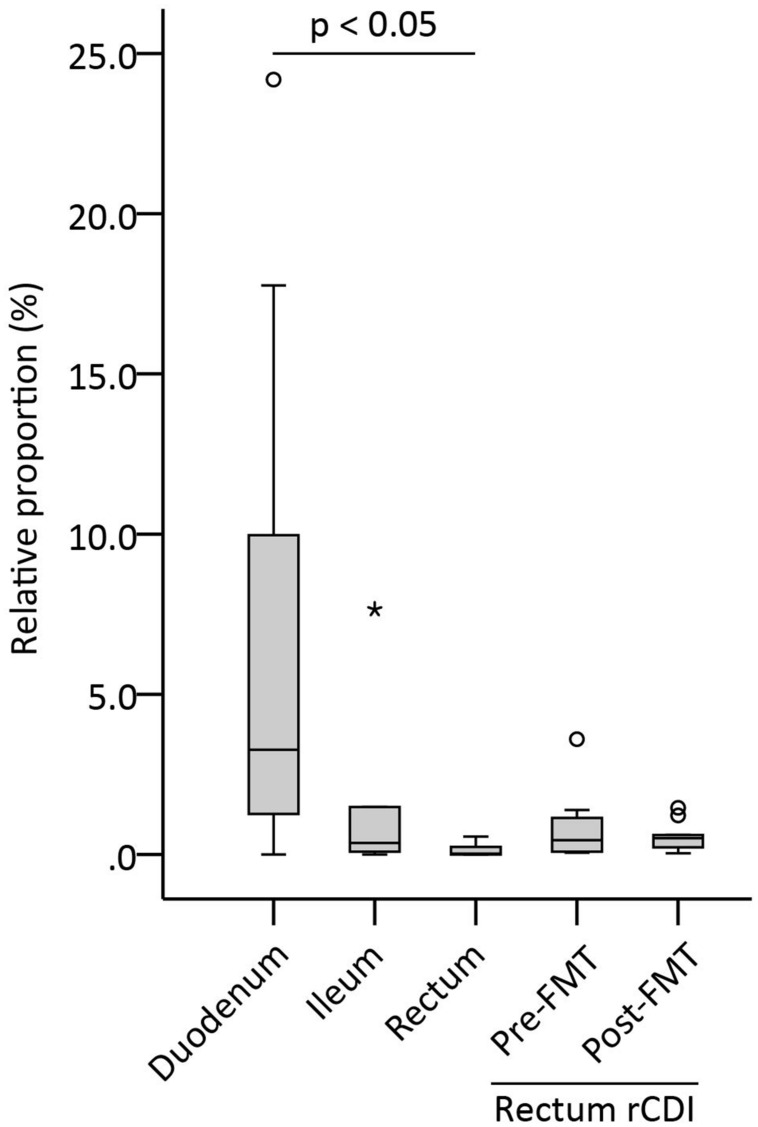
**Relative proportion of *Sutterella* spp. (% of all bacteria) in different parts of the intestinal mucosa (duodenum, ileum, rectum) in healthy adults and in patients pre- and post-FMT.** Duodenum (*n* = 17), ileum (*n* = 6), and rectum (*n* = 5) biopsies were obtained from the healthy subjects. Other rectum samples (*n* = 11) were collected from the patients who had undergone a successful fecal microbiota transplantation (FMT) for recurrent *Clostridium difficile* infection (rCDI). Biopsies obtained from the rCDI patients were collected pre-FMT and 1 month after FMT. A circle and an asterisk indicate outliers greater than 1.5 and 3 times the interquartile range, respectively.

In order to assess the amount of *Sutterella* spp. in the different parts of a healthy intestine, mucosal biopsies from duodenum, ileum, and rectum were analyzed with *Sutterella*-specific and universal qPCR assays. The results were calculated as a relative proportion of *Sutterella* spp. compared to all bacteria (**Figure 1**). Adult patients who underwent successfully the FMT therapy for a rCDI were also included in the study and rectal samples both pre- and post-FMT were analyzed. We observed a decreasing gradient of *Sutterella* spp. along the GI tract from the small intestine to the large bowel. The relative abundance of *Sutterella* in the duodenum and ileum was significantly higher (*p* = 0.005) as compared to the rectum, while the percentage of these species of the whole duodenal microbiota seemed to vary greatly between individuals. The pre- and post-FMT samples of rCDI patients (*p* = 0.443) or the samples from rCDI patients and other study subjects did not differ in their *Sutterella* abundance (*p* = 0.107; **Figure 1**). A gradient in the relative abundance of *Sutterella* spp. along the GI tract was observed also at individual level in the case of one subject, from whom biopsies of all three intestinal locations were obtained. In these samples *Sutterella* spp. constituted 1.3, 0.3, and 0.2% of the total microbiota in the duodenum, ileum, and rectum, respectively.

### No Difference in *Sutterella* Prevalence between Pediatric CeD and IBD Patients and Healthy Children

Next, we studied whether *Sutterella* spp. and specifically *S. wadsworthensis* are differently present in the duodenum or colon of pediatric patients with CeD or IBD compared to the healthy controls. The 16S rRNA gene copy numbers in these samples were too low for reliable quantitation and, therefore, only qualitative analysis could be performed. The PCR analysis showed that *Sutterella* spp. were present in half of healthy children’s duodenum and in six out of nine CeD patients (*p* = 0.622; **Table 1**). Surprisingly, *S. wadsworthensis* was not detected in the duodenal biopsies of either group. The biopsies from pediatric UC or CD patients and the controls were obtained from different parts of the colon (Supplementary Table S2). The presence of *Sutterella* spp. was identified in >80% of the patients in all three groups (*p* = 1.000), whereas the prevalence of *S. wadsworthensis* was 29–40% (*p* = 0.799). Furthermore, there was no statistical difference in the prevalence of *Sutterella* spp. or *S. wadsworthensis* when comparing the UC and CD patients and the healthy controls or depending on the intestinal biopsy location.

**Table 1:** The prevalence of *Sutterella* spp. and *S. wadsworthensis* in the intestinal mucosa of the pediatric subjects.

	Studied subjects	
	*Sutterella* spp.	*S. wadsworthensis*
**Duodenum**		
Healthy controls	3/6	ND
Celiac disease	6/9	ND
**Colon**		
Healthy controls	22/27	9/27
Ulcerative colitis	21/25	10/25
Crohn’s disease	12/14	4/14

*ND, not detected.*

### *Sutterella wadsworthensis* Shows the Highest Adhesion to Mucus and Extracellular Matrix Proteins

**FIGURE 2:**
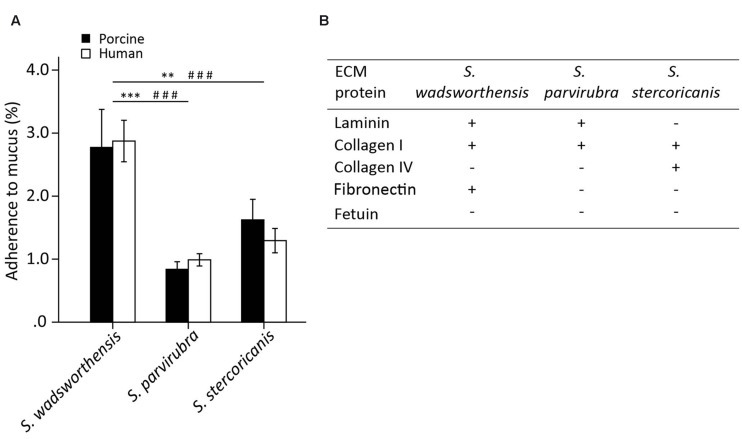
**Adherence of *Sutterella* spp. to porcine and human mucus and human extracellular matrix (ECM) proteins. (A)** Mucus adhesion data is presented as an average adhesion percentage of the added bacteria and error bars show the standard deviation between four technical replicates (parallel wells) from a representative experiment. Asterisks indicate a statistically significant difference between the species in porcine mucus binding (^∗∗^*p* < 0.01, ^∗∗∗^*p* < 0.001) and # symbol in human mucus binding (^###^*p* < 0.001). **(B)** Adherence to ECM proteins compared to the background binding (BSA). Fetuin as a highly glycosylated protein was used as control for non-specific binding. Four technical replicates were used in each biological replicate and the adhesion percentages were converted to +/- scaling depending on the statistical significance. Symbol “+” indicates a significantly higher binding as compared to the background (BSA, *p* < 0.05) and symbol “-” signifies a statistically non-significant binding as compared to the background.

We examined the adherence of *S. wadsworthensis*, *S. parvirubra*, and *S. stercoricanis* to immobilized human and porcine mucus, as well as to ECM proteins, including type I and IV collagens, laminin, and fibronectin. The adherence ability of *S. wadsworthensis* to porcine mucus was better compared to the other two species (**Figure 2A**). In fact, *S. parvirubra* did not bind mucus, since less than 1% of the added cells had adhered which is considered to be non-specific background binding. The same differences between *S. wadsworthensis* and the other species were also observed in the adhesion to human mucus (**Figure 2A**). *Sutterella* spp. showed no difference between human and porcine mucus binding. When studying the adherence of these bacteria to ECM proteins, fetuin as a highly glycosylated protein and BSA as a non-glycosylated protein were included in the assay as controls. *S. wadsworthensis* showed significantly higher adhesion to laminin, collagen I, and fibronectin compared to the background-level (BSA; **Figure 2B**). Moreover, its adherence to BSA was 2.3%, whereas the same adhesion ratios for *S. parvirubra* and *S. stercoricanis* were merely 0.8 and 1.5%, respectively (data not shown). *S. parvirubra* could only bind to laminin and collagen I, and *S. stercoricanis* to collagens I and IV (**Figure 2B**).

### *Sutterella parvirubra* Adheres Best to Differentiated Enterocytes and Can Displace *S. wadsworthensis*

**FIGURE 3:**
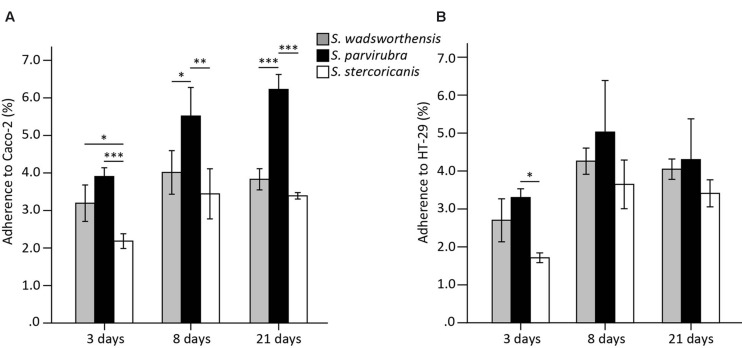
**Adherence of *Sutterella* spp. to Caco-2 (A) and HT-29 (B) cell lines at different growth stages (3, 8, or 21 days post-plating).** Data is shown as means of adhered bacteria from the added bacteria (%) and standard deviations of four technical replicates (parallel wells) from a representative experiment. Asterisks indicate significant difference in adhesion capability between the species: ^∗^*p* < 0.05, ^∗∗^*p* < 0.01, ^∗∗∗^*p* < 0.001.

To compare the adhesion capability of *Sutterella* spp. to IECs, we used the Caco-2 and HT-29 cell lines as models of enterocytes. Caco-2 cells start to differentiate 5–7 days post-plating and both cell lines lack the mucus layer covering the intestine. Cells at three different growth stages were included in the assays: undifferentiated (3 days post-plating) as well as 8- and 21 (partially and fully differentiated)-day-old cells (Park et al., 2015). HT-29 cells were grown similarly for comparison, although they do not differentiate. In general, *Sutterella* spp. adhered equally well to both cell lines. The adherence level of *S. parvirubra* and *S. stercoricanis* was lower to the undifferentiated Caco-2 cells (3 days; **Figure 3**). Interestingly, *S. parvirubra* adhered significantly better to 8- and 21-day-old Caco-2 cells compared to the other two species. There was no difference in the adhesion of all three species to HT-29 cells except *S. parvirubra* adhered better than *S. stercoricanis* to 3-day-old cells. In the displacement, exclusion, and competition assays, the effect of *S. parvirubra* on the adherence of *S. wadsworthensis* was tested using 8-day-old cell lines. The adhesion was significantly lowered (*p* < 0.001) to 55 and 52% in competition to Caco-2 and HT-29 cells, respectively (**Table 2**). Furthermore, displacement by *S. parvirubra* reduced the binding of *S. wadsworthensis* to both enterocyte lines (*p* < 0.05) but the effect was more profound in Caco-2 cells (73%). The preliminary results on the capability of *S. parvirubra* to exclude *S. wadsworthensis* showed merely a 15% reduction in the adherence to Caco-2 cells and no effect on the adherence to HT-29 cells (data not shown).

**Table 2:** Adherence of *S. wadsworthensis* to 8-day-old Caco-2 and HT-29 cells in the competition and displacement assays.

Assays	Cell lines	
	Caco-2	HT-29
No competition	100%	100%
Competition by *S. parvirubra*	55 ± 11%^∗∗∗^	52 ± 10%^∗∗∗^
Displacement by *S. parvirubra*	73 ± 5%^∗^	80 ± 11%^∗^

*The results (mean ± SD of four technical replicates from a representative experiment) are presented as percentages compared to the adhesion of S. wadsworthensis without competition (100%). Asterisks indicate a significantly reduced adhesion of S. wadsworthensis. ^∗^p < 0.05, ^∗∗∗^p < 0.001.*

### *Sutterella* spp. Have no Effect on Epithelial Barrier Function *In vitro*

**FIGURE 4:**
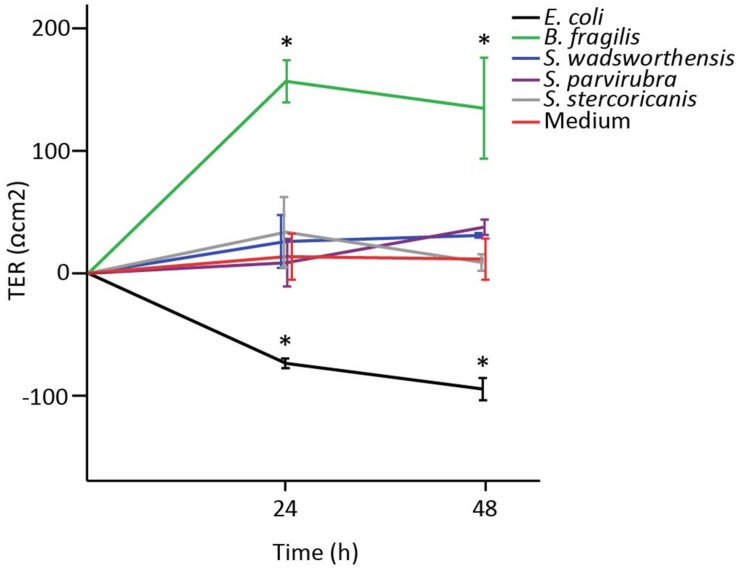
**The effect of *Sutterella* spp. on transepithelial electrical resistance (TER) of Caco-2 monolayer.** *E. coli* was used as a negative control and *B. fragilis* as a positive control. Results from a representative experiment are shown as mean TER values (Ωcm^2^) and standard deviations of three technical replicates (parallel wells). An asterisk indicates significantly different (^∗^*p* < 0.05) values between the bacteria-treated and the untreated (only growth medium) Caco-2 monolayers.

In order to study the impact of *Sutterella* spp. on the epithelial barrier function, we measured TER of a Caco-2 monolayer co-cultured with the bacteria. TER of 8-day-old Caco-2 cells (5 days after confluence) was measured at 24 h intervals for 48 h in total. Partially differentiated Caco-2 monolayer was used to imitate the intestinal conditions comprising of enterocytes still in the process of maturation and prone to changes of barrier integrity due to external stimuli (Reunanen et al., 2015; Valenzano et al., 2015). *Sutterella* spp. had no effect on the epithelial barrier function as TER values remained at the same level as the values of cells treated with culture medium (**Figure 4**). On the contrary, already within 24 h *B. fragilis* (positive control) had greatly enhanced the monolayer integrity, whereas *E. coli* (negative control) had significantly decreased it.

### *S. wadsworthensis* Induces a Moderate IL-8 Response in Enterocytes

**FIGURE 5:**
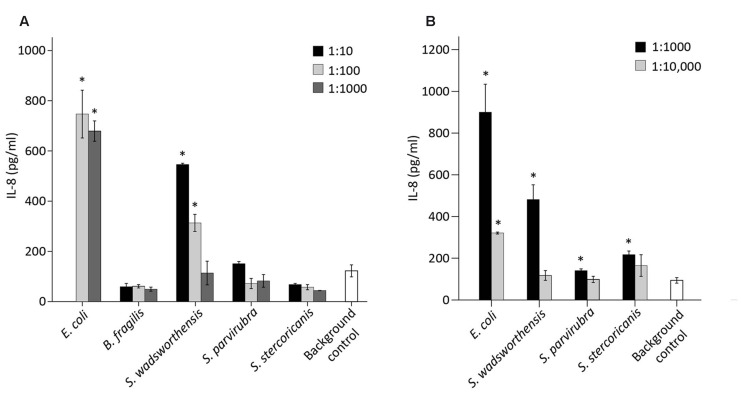
**IL-8 production in HT-29 cells induced by bacterial cell suspensions and LPS preparations. (A)** IL-8 response in HT-29 cells by *S. wadsworthensis*, *S. parvirubra*, *S. stercoricanis*, *B. fragilis* (positive control), and *E. coli* (negative control) with 1:10, 1:100, and 1:1,000 dilutions from OD_600__nm_ 0.25 adjusted cell suspensions. The 1:10 dilution is not shown for *E. coli* due to excessive toxicity to the HT-29 cells. **(B)** IL-8 production induced by 1:1,000 and 1:10,000 dilutions of LPS preparations from *E. coli* and *Sutterella* spp. LPS preparations were made from the OD_600__nm_ 0.25 normalized cell suspensions and the dilutions were thereof. Results from the representative experiment are shown as means and standard deviations of three technical replicates (parallel wells). The culture medium was used as a background control in both assays. An asterisk indicates a significant (^∗^*p* < 0.05) IL-8 production above the background level.

We examined the potential pro-inflammatory properties of *Sutterella* spp. by incubating 10-fold dilutions of the bacteria with HT-29 monolayer followed by measurement of IL-8 release from the cells. Again, *E. coli* and *B. fragilis* were used as controls, whereas the cells incubated solely with the culture medium functioned as a background control. For *E. coli*, the results of 1:10 dilution are not shown due to excess stimulation. Previously *B. fragilis* has been shown not to induce an IL-8-response in HT-29 cells (Reunanen et al., 2015). Similarly, *S. parvirubra* and *S. stercoricanis* did not have any effect on the IL-8 production as levels were lower than in the medium control (**Figure 5A**). *E. coli* and *S. wadsworthensis*, on the other hand, induced a significant, dose-dependent IL-8 response in the HT-29 cells, although for *S. wadsworthensis* the effect was only reached with 1:10 and 1:100 dilutions.

The same assay was carried out with crude LPS preparations of *Sutterella* spp. and *E. coli*. All three *Sutterella* spp. induced an IL-8 production above the medium control level with 1:1,000 dilution but not with 1:10,000 dilution (**Figure 5B**). The effect was stronger with the LPS preparation from *S. wadsworthensis*. The same LPS preparations were run in SDS-PAGE gel and visualized with silver staining to compare the LPS structures (Supplementary Figure S1A). All *Sutterella* spp. differed in their LPS polysaccharide chain profiles. The short side-chains expressed by *S. stercoricanis* were clearly visible as a ladder formation. The antigenic similarity of LPS structures between *Sutterella* spp. and *E. coli* was tested using whole-cell lysates and a rabbit polyclonal antiserum against *E. coli* LPS. The antiserum only reacted with *E. coli* lysate (Supplementary Figure S1B). Further, we compared the possible LPS differences between *Sutterella* spp. and *E. coli* by surveying the available genomic data for *S. parvirubra* and *S. wadsworthensis* using the NCBI (The National Center for Biotechnology Information, USA) database and BLAST (Basic Local Alignment Search Tool, blastp algorithm) for genes that encode enzymes for Kdo_2_-lipid A modification. We found out that *Sutterella* spp. lack the genes for LpxL and LpxM acyltransferases, which are present in *E. coli* (**Table 3**).

**Table 3:** Comparison of Kdo_2_-lipid A biosynthesis enzymes between *E. coli* K12 and *Sutterella* spp.

Organism	Genome accession number	Enzymes needed for Kdo_2_-lipid A biosynthesis								
		LpxA	LpxC	LpxD	LpxH	LpxB	LpxK	WaaA	LpxL	LpxM
*Escherichia coli* K12	NC_000913 (Blattner et al., 1997)	+	+	+	+	+	+	+	+	+
*Sutterella wadsworthensis* 2_1_59BFAA	ADMG00000000.1 (Earl et al., unpublished)	+	+	+	+	+	+	+		
*Sutterella parvirubra* YIT11816	AFBQ00000000.1 (Weinstock et al., unpublished)	+	+	+	+	+	+	+		

*LpxA, UDP-N-acetylglucosamine acetyltransferase; LpxC, UDP-3-O-[3-hydroxymyristoyl] N-acetylglucosamine deacetylase; LpxD, UDP-3-O-(3-hydroxymyristoyl)glucosamine N-acyltransferase; LpxH, UDP-2,3-diacylglucosamine pyrophosphohydrolase; LpxB, lipid-A-disaccharide synthase; LpxK, lipid A 4′-kinase; WaaA, 3-deoxy-D-manno-octulosonate lipid A transferase; LpxL, Kdo2-lipid IVa lauroyl-ACP acyltransferase; LpxM, Kdo2-lauroyl-lipid IVa-myristoyl-ACP acyltransferase*.

## Discussion

Our findings show that the relative abundance of *Sutterella* spp. is substantial in the duodenum of adults with a decreasing gradient toward the colon, which has not been demonstrated before. The bile resistant and microaerophilic properties of *Sutterella* spp. (Wexler et al., 1996; Greetham et al., 2004) do indeed imply they are adapted to the physiological conditions of the small intestine, which, compared to the colon, consist of bile acids secreted from the bile duct, lower pH and higher levels of antimicrobial peptides and oxygen (Donaldson et al., 2016). The harsh environment of the small intestine naturally influences the microbiota composition resulting in lower bacterial density and diversity, as well as in the enrichment of certain bacteria, such as Proteobacteria (Zoetendal et al., 2012; Cheng et al., 2013; Li et al., 2015). According to Li et al. (2015), the abundance of Proteobacteria in biopsies and luminal contents of the duodenum in healthy adults varied between 33 and 39%, whereas the proportion in the rectal biopsies and feces was merely 5–12%. Moreover, similar to our results, they reported that *Sutterella* was one of the dominant genera only in the duodenal biopsies. In this study, we also examined whether *Sutterella* spp. contribute to the dysbiotic microbiota of rCDI patients and studied their relative abundance pre-FMT (dysbiotic state) and after a successful FMT treatment of rCDI. Before FMT, rCDI patients have a dysbiotic microbiota with a higher abundance of Proteobacteria and facultative anaerobes, which are decreased to normal level by FMT together with an increase of obligately anaerobic gut commensals (van Nood et al., 2013). We did not observe a statistically significant difference in the relative proportion of *Sutterella* before and after FMT and, thus, the genus does not seem to contribute to rCDI-associated dysbiosis.

In accordance with the previous studies (Mukhopadhya et al., 2011; Hansen et al., 2013), we found no difference in the prevalence of *Sutterella* spp. or *S. wadsworthensis* between the healthy controls and the pediatric IBD or CeD patients. These results reinforce the suggestion that *Sutterella* spp. are likely to be commensals in the human GI tract and do not have a role in the etiopathology of these diseases. In agreement with this view, *Sutterella* has been associated, as a sole genus from Proteobacteria, with a healthy outcome after ileal pouch-anal anastomosis due to UC (Tyler et al., 2013; Morgan et al., 2015). Furthermore, a lower abundance of *Sutterella* in patients with new-onset CD (Gevers et al., 2014) or non-alcoholic fatty liver disease has been shown recently (Del Chierico et al., 2016).

The adherence of bacteria to mucus and IECs is considered crucial in terms of colonization and interaction with the intestinal mucosa, especially in the small intestine, where transit time is shorter and, thus, an efficient binding ability is of greater importance (Donaldson et al., 2016). A mucus layer, composed of gel-forming intestinal mucin MUC2, partially or completely covers the small intestine and the colon creating a barrier between the epithelium and the bacterial community (Johansson et al., 2013; Pelaseyed et al., 2014; Donaldson et al., 2016). However, unlike the colon with a dense inner and a loose outer layer, the mucus covering the small intestine is loose and unattached, allowing bacteria to penetrate and interact with IECs (Johansson et al., 2013). ECM components, on the other hand, are located in the basal lamina providing support for the epithelial cells and exposed to the luminal bacteria only if the mucosal surface is disrupted (Westerlund and Korhonen, 1993).

In this study we observed that *S. wadsworthensis* bound markedly better to mucus and ECM proteins than the other two species, whereas *S. parvirubra* could adhere at a higher level to differentiated enterocytes and displace *S. wadsworthensis* in adhesion. *Sutterella* spp. adhered equally well or even better to enterocytes than the well-known probiotic strain *Lactobacillus rhamnosus* GG or commensal mucosa-associated bacterium *Akkermansia muciniphila* by using the same assay indicating a moderate adhesion ability (Reunanen et al., 2015). A direct contact with the host tissue by *Sutterella* spp. is likely, since the thinner mucus layer of the small intestine is discontinuous enabling a direct interaction between the microbiota and the IECs (Johansson et al., 2013), and considering the abundance of these species in the duodenum. Therefore, *Sutterella* spp. have an opportunity to interact actively with the host. Gut commensals are needed to induce the steady-state Th17 differentiation (Gaboriau-Routhiau et al., 2009; Ivanov et al., 2009; Atarashi et al., 2015). Importantly, this immunostimulatory action was recently shown to be dependent on the ability of bacteria to adhere to IECs, which then cue Th17 differentiation in LP (Atarashi et al., 2015). Consequently, by adhering to IECs *Sutterella* spp. may participate in such action. We used three type strains to examine the adherence and the immunostimulatory abilities of the genus *Sutterella* and to our knowledge, this is the first study to explore the epithelial interactions of *Sutterella* spp. However, we acknowledge that further studies by using fresh clinical isolates from patients and healthy individuals are needed to confirm our findings on the commensal nature of *Sutterella* spp.

IECs secrete a cytokine IL-8 in response to various inflammatory stimuli activating neutrophils into the infected mucosa (Lammers et al., 1994; Mitsuyama et al., 1994). We observed that *S. wadsworthensis* caused stronger inflammatory cascade in the enterocytes than the other two species by eliciting an IL-8 response, although the stimulation was merely moderate compared to the IL-8 release induced by *E. coli*. However, none of *Sutterella* spp. had an impact on the epithelial barrier function indicating their role as harmless bystanders. Furthermore, the mild pro-inflammatory capacity of *S. wadsworthensis* without the disruption of epithelial integrity may assist in keeping the host’s immune system alerted at an appropriate, physiological level. In another study, *S. wadsworthensis* strains isolated from healthy controls and IBD patients induced higher levels of TNF-α compared to *E. coli* in human monocytes, but no difference in the cytokine stimulation was detected between the strains isolated from the two groups (Mukhopadhya et al., 2011).

We found that there are differences in the pro-inflammatory properties between *Sutterella* spp. as well as between *Sutterella* spp. and *E. coli* and, thus, speculate that this could be due to their varying LPS structures. LPS on the surface of Gram-negative bacteria is composed of Kdo_2_-Lipid A (endotoxin), core-oligosaccharide and O-antigen repeats and recognized by the host TLR4–MD2 complex (Miller et al., 2005; Han et al., 2013; Polissi and Sperandeo, 2014). We visualized the differences in the LPS structure between the species by silver staining of the SDS-PAGE separated LPS, which revealed differences in O-antigen repeats, and by immunoblotting with an antiserum against *E. coli* LPS, which did not recognize LPS from *Sutterella* spp. However, the endotoxicity of LPS is more determined by the lipid A structure, such as phosphorylation degree as well as the number and length of acyl chains, which affects its ligand affinity to TLR4–MD2 (Kobayashi et al., 2006; Park et al., 2009; Han et al., 2013). The *E. coli* lipid A is hexa-acylated with two phosphate groups and therefore can activate the TLR4–Mal–MyD88 pathway making it highly virulent, whereas the penta-acylated or monophosphorylated forms of lipid A are 100-fold less toxic by signaling through TLR4–TRAM–TRIF (Park et al., 2009; Herath et al., 2011; Han et al., 2013).

We next searched the genomes of *S. wadsworthensis* and *S. parvirubra* (NCBI database) and observed that these species lack the genes for LpxL and LpxM, which are the enzymes responsible for adding the secondary acyl chains (hexa-acylation), and, consequently, are likely to carry the tetra-acylated lipid A with reduced toxicity. Our results support this view, since *Sutterella* LPS did not produce a strong IL-8 response in the HT-29 cells in contrast to *E. coli*. Quite surprisingly only *E. coli* and related Proteobacteria have all nine genes needed for the complete Kdo_2_-lipid A biosynthesis (Opiyo et al., 2010). Recently, it was shown that the LPS of *Bacteroides dorei*, which has tetra- and penta-acylated lipid A, differs majorly in its immunomodulation capacity from that of *E. coli* LPS (Vatanen et al., 2016). The structural diversity of LPS and lipid A among Gram-negative bacteria and Proteobacteria is wide and, therefore, it is essential to study the host–microbe interactions of a vast repertoire of intestinal bacteria. Our study expands such studies to cover *Sutterella* spp.

It is of interest that *in vitro Sutterella* spp. can be successfully grown only on solid media or in chopped meat broth, which includes solid particles (Wexler et al., 1996; Mukhopadhya et al., 2011; Williams et al., 2012; Hansen et al., 2013). This may reflect their life-style in the GI track, i.e., that they are mainly epithelium-associated bacteria. Based on our adhesion results, we speculate that *Sutterella* spp. possess specific niches in the small intestine. *S. parvirubra* adhered strongest to enterocytes and showed competitiveness against *S. wadsworthensis* in enterocyte binding, whereas *S. wadsworthensis* displayed the strongest binding to intestinal mucus and ECM proteins. The human GI tract can be colonized by single or multiple *Sutterella* spp. (Williams et al., 2012). Considering the host–microbiota symbiosis, it might be preferable that *S. parvirubra* with low pro-inflammatory capacity is tissue-associated and *S. wadsworthensis* with the ability of inducing inflammation cascades occupies the mucus layer. Proteobacteria most likely are involved in the pro-inflammatory changes leading to dysbiosis in the human GI tract (Mukhopadhya et al., 2012; Shin et al., 2015). Yet, it is the largest and most diverse phylum comprising, for instance, IBD associated Enterobacteriaceae and other medically important gammaproteobacteria, as well as Burkholderiales order belonging to Betaproteobacteria, which have not been linked directly to any GI diseases (Mukhopadhya et al., 2012). However, *Sutterella* spp. do not seem to contribute to a dysbiotic state of the gut, but instead their presence, especially in the duodenum, and ability to adhere to IECs indicate them to be mutualistic keeping the immune system alerted.

In conclusion, our results indicate that the members of the genus *Sutterella* are widely prevalent commensals with IEC-adhesion and mild pro-inflammatory capacities in the human GI tract.

## Author Contributions

KH, RS, and VK conceived the study. KH and VK performed the experiments. KH and RS interpreted the data and wrote the manuscript. MK and PA recruited patients as well as collected clinical samples and data. All authors critically revised the manuscript for intellectual content and approved the final version of the manuscript.

## Conflict of Interest Statement

The authors declare that the research was conducted in the absence of any commercial or financial relationships that could be construed as a potential conflict of interest.
