# Supplementary material for: Mucosal Prevalence and Interactions with the Epithelium Indicate Commensalism of Sutterella spp
Source: Front Microbiol. 2016 Oct 26;7:1706. doi: 10.3389/fmicb.2016.01706 (PMC5080374; doi:10.3389/fmicb.2016.01706)
Supplement: Supplementary file 1 [file Data_Sheet_1.PDF]

Supplementary Material

Mucosal prevalence and interactions with the epithelium indicate commensalism of *Sutterella* spp.

Kaisa Hiippala*, Veera Kainulainen, Marko Kalliomäki, Perttu Arkkila, Reetta Satokari

*** Correspondence:** Kaisa Hiippala: kaisa.hiippala@helsinki.fi

1 Supplementary Figures

A **B**

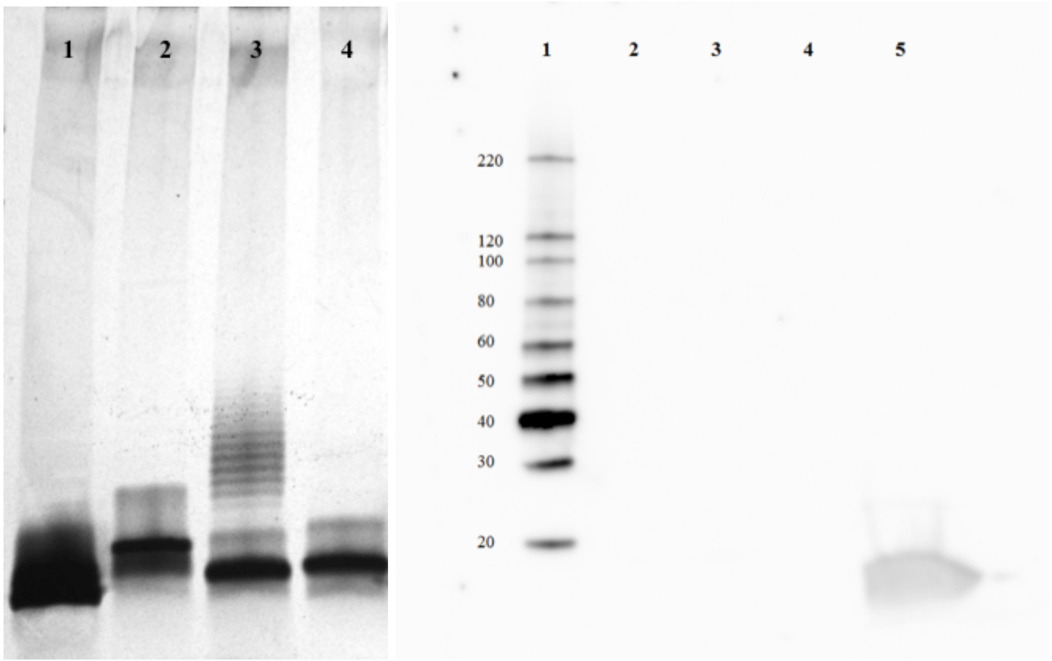

Supplementary Figure 1. (A) Visualization of LPS structure of *E. coli* (lane 1), *S. wadsworthensis* (lane 2), *S. stercoricanis* (lane 3) and *S. parvirubra* (lane 4) on SDS-PAGE with silver staining. **(B)** Western blotting of whole-cell lysates of *S. wadsworthensis* (lane 2), *S. stercoricanis* (lane 3), *S. parvirubra* (lane 4) and *E. coli* (positive control, lane 5) using a rabbit polyclonal antiserum against *E. coli* LPS.

2 Supplementary Tables

Supplementary Table 1. Characteristics of adult patients and biopsy location.

Subject	Gender	Age	Biopsy location	Group
1	F	73	duodenum	Healthy
2	F	61	duodenum	Healthy
3	F	73	duodenum	Healthy
4	F	85	duodenum	Healthy
5	M	81	duodenum	Healthy
6	F	85	duodenum	Healthy
7	F	43	duodenum	Healthy
8	F	47	duodenum	Healthy
9	F	53	duodenum	Healthy
10	F	80	duodenum	Healthy
11	F	59	duodenum	Healthy
12	F	73	duodenum	Healthy
13	F	59	duodenum	Healthy
14	F	71	duodenum	Healthy
14			ileum	Healthy
14			rectum	Healthy
15	F	30	duodenum	Healthy
16	F	65	ileum	Healthy
16			rectum	Healthy
17	F	68	ileum	Healthy
17			rectum	Healthy
18	M	73	duodenum	Healthy
19	F	46	duodenum	Healthy
20	M	73	ileum	Healthy
20			rectum	Healthy
21	M	56	ileum	Healthy
22	M	66	ileum	Healthy
22			rectum	Healthy
23	M	67	rectum	Pre-FMT
23			rectum	Post-FMT
24	M	81	rectum	Pre-FMT
24			rectum	Post-FMT
25	F	58	rectum	Pre-FMT
25			rectum	Post-FMT
26	F	44	rectum	Pre-FMT
26			rectum	Post-FMT
27	F	31	rectum	Pre-FMT
27			rectum	Post-FMT
28	F	20	rectum	Pre-FMT
28			rectum	Post-FMT

29	F	57	rectum	Pre-FMT
29			rectum	Post-FMT
30	M	63	rectum	Pre-FMT
30			rectum	Post-FMT
31	F	82	rectum	Pre-FMT
31			rectum	Post-FMT
32	F	81	rectum	Pre-FMT
32			rectum	Post-FMT
33	M	45	rectum	Pre-FMT
33			rectum	Post-FMT

Supplementary Table 2. Characteristics of pediatric patients, biopsy location and disease activity.

Subject	Gender	Age	Biopsy location	Group	Disease activity
1	M	7	duodenum	Healthy	
2	F	11	duodenum	Celiac disease	Newly diagnosed before implementation of a gluten-free diet
3	M	3	duodenum	Celiac disease	Newly diagnosed before implementation of a gluten-free diet
4	F	16	duodenum	Healthy	
5	M	5	duodenum	Celiac disease	Newly diagnosed before implementation of a gluten-free diet
6	F	11	duodenum	Healthy	
7	M	9	duodenum	Celiac disease	Newly diagnosed before implementation of a gluten-free diet
8	M	9	duodenum	Healthy	
9	F	13	duodenum	Celiac disease	Newly diagnosed before implementation of a gluten-free diet
10	F	13	duodenum	Celiac disease	Newly diagnosed before implementation of a gluten-free diet
11	M	7	duodenum	Celiac disease	Newly diagnosed before implementation of a gluten-free diet
12	F	5	duodenum	Healthy	
13	F	14	duodenum	Celiac disease	Newly diagnosed before implementation of a gluten-free diet
14	F	12	duodenum	Healthy	
15	F	10	duodenum	Celiac disease	Newly diagnosed before implementation of a gluten-free diet
16	M	11	colon, cecum	Ulcerative colitis	Active disease
17	F	12	colon, descending	Ulcerative colitis	Active disease
18	F	17	colon, asceding	Ulcerative colitis	Active disease
19	M	12	colon, cecum	Ulcerative colitis	In remission
20	M	7	colon, cecum	Healthy	
21	F	12	colon, descending	Healthy	
22	M	17	colon, sigmoid	Crohn's disease	
23	F	12	colon, ascending	Ulcerative colitis	Active disease
24	M	4	colon, descending	Healthy	
25	F	4	colon, cecum	Healthy	
26	M	18	colon, descending	Crohn's disease	Active disease
27	F	11	colon, descending	Ulcerative colitis	Active disease
28	M	15	colon, sigmoid	Ulcerative colitis	In remission
29	M	12	colon, sigmoid	Healthy	
30	F	9	colon,descending	Healthy	
31	M	16	colon, aescending	Ulcerative colitis	Active disease
32	F	7	colon, descending	Healthy	
33	M	14	colon, sigmoid	Ulcerative colitis	Active disease
34	F	18	colon, descending	Ulcerative colitis	Active disease
35	M	13	colon, descending	Crohn's disease	In remission
36	F	15	colon, descending	Healthy	

37	M	14	colon, sigmoid	Healthy	
38	F	11	colon, descending	Healthy	
39	M	16	colon, sigmoid	Ulcerative colitis	Active disease
40	M	4	colon, cecum	Healthy	
41	F	12	colon, aescending	Healthy	
42	M	10	colon, aescending	Crohn's disease	In remission
43	M	6	colon, descending	Healthy	
44	M	5	colon, aescending	Healthy	
45	M	17	colon, aescending	Ulcerative colitis	Active disease
46	M	16	colon, cecum	Ulcerative colitis	In remission
47	M	10	colon, aescending	Ulcerative colitis	In remission
48	M	15	colon, cecum	Crohn's disease	Active disease
49	F	15	colon, aescending	Ulcerative colitis	In remission
50	F	13	colon, aescending	Ulcerative colitis	Active disease
51	M	14	colon, aescending	Crohn's disease	Active disease
52	M	16	colon, aescending	Ulcerative colitis	Active disease
53	M	13	colon, aescending	Crohn's disease	In remission
54	M	13	colon, aescending	Crohn's disease	In remission
55	F	13	colon, descending	Ulcerative colitis	Active disease
56	F	7	colon, aescending	Ulcerative colitis	Active disease
57	M	14	colon, cecum	Ulcerative colitis	In remission
58	F	15	colon, aescending	Ulcerative colitis	In remission
59	M	3	colon, cecum	Healthy	
60	M	3	colon, cecum	Crohn's disease	In remission
61	F	5	colon, aescending	Ulcerative colitis	Active disease
62	M	15	colon, cecum	Crohn's disease	In remission
63	M	12	colon, cecum	Ulcerative colitis	Active disease
64	M	14	colon, cecum	Crohn's disease	Active disease
65	F	11	colon, cecum	Healthy	
66	M	10	colon, cecum	Healthy	
67	M	14	colon, cecum	Ulcerative colitis	Active disease
68	F	10	colon, cecum	Healthy	
69	M	3	colon, cecum	Crohn's disease	Active disease
70	F	7	colon, cecum	Healthy	
71	M	8	colon, cecum	Crohn's disease	Active disease
72	F	15	colon, cecum	Ulcerative colitis	In remission
73	M	11	colon, cecum	Healthy	
74	F	3	colon, cecum	Healthy	
75	M	2	colon, cecum	Healthy	
76	F	8	colon, cecum	Healthy	
77	M	4	colon, cecum	Healthy	
78	M	15	colon, cecum	Crohn's disease	Active disease
79	M	12	colon, cecum	Healthy	
80	M	2	colon, cecum	Healthy	

81	M	11	colon, cecum	Healthy
